# Establishment of an animal model of a pasteurized bone graft, with a preliminary analysis of muscle coverage or FGF-2 administration to the graft

**DOI:** 10.1186/1749-799X-4-31

**Published:** 2009-08-04

**Authors:** Tatsuya Yoshida, Akio Sakamoto, Nobuaki Tsukamoto, Koichi Nakayama, Yukihide Iwamoto

**Affiliations:** 1 Department of Orthopaedic Surgery, Graduate School of Medical Sciences, Kyushu University, Fukuoka, Japan

## Abstract

**Background:**

Pasteurized bone grafting is used following the excision of a bone tumor for the purpose of eliminating neoplastic cells while preserving bone-inducing ability. In the hopes of guaranteeing the most favourable results, the establishment of an animal model has been urgently awaited. In the course of establishing such a model, we made a preliminary examination of the effect of muscle coverage or fibroblast growth factor 2 (FGF-2) administration radiographically.

**Methods:**

Forty pasteurized intercalary bone grafts of the Wistar rat femur treated at 60°C for 30 min were reimplanted and stabilized with an intramedullary nail (1.1 mm in diameter). Some grafts were not covered by muscle after the implantation, so that they could act as a clinical model for wide resection, and/or these were soaked with FGF-2 solution prior to implantation. The grafts were then divided into 3 groups, comprising 12 grafts with muscle-covering but without FGF-2 (MC+; FGF2-), 12 grafts without muscle-covering and without FGF-2 (MC-; FGF2-) and 16 grafts without muscle covering but with FGF-2 (MC-; FGF2+).

**Results:**

At 2 weeks after grafting, the pasteurized bone model seemed to be successful in terms of eliminating living cells, including osteocytes. At 4 weeks after grafting, partial bone incorporation was observed in half the (MC+; FGF2-) cases and in half the (MC-; FGF2+) cases, but not in any of the (MC-; FGF2-) cases. At 12 weeks after grafting, bone incorporation was seen in 3 out of 4 in the (MC+; FGF2-) group (3/4: 75%) and in 3 out of 8 in the (MC-; FGF2+) group (3/8: 38%). However, most of the grafted bones without FGF-2 were absorbed in all the cases, massively, regardless of whether there had been muscle-covering (MC+; FGF2-; 4/4: 100%) or no muscle-covering (MC-; FGF2-; 4/4: 100%), while bone absorption was noted at a lower frequency (2/8: 25%) and to a lower degree in the (MC-; FGF2+) group.

**Conclusion:**

In conclusion, we have established an animal pasteurized bone graft model in rats. Pasteurized bone was able to maintain bone induction ability. Despite the low number of cases in each group, the results of each group suggest that muscle-covering has an effect on bone incorporation, but that it is not able to prevent bone absorption to the pasteurized bone. However, an application of FGF-2 may have a positive effect on bone incorporation and may be able to prevent bone absorption of the graft in cases of pasteurized bone graft.

## Background

Pasteurized bone grafting is a method of heating an excised bone at a low temperature [1], such as at 60°C for 30 min [2], for the purpose of eliminating neoplastic cells. This method can be used for reconstruction after the resection of bone and soft-tissue tumors [3,4]. Pasteurized bone is reported to preserve bone induction ability, and to act as scaffolding for invasion by viable bone tissue with progressive substitution from peripheral adjacent bone, resulting in deposition of new bone on the graft matrix [5]. Other advantages of the method include a precise anatomical fit, and no risk of disease transmission or immunological reaction [4-10]. Regardless of such advantages, clinical problems, such as over-absorption of the grafted bone or infection, may be due to the prolonged existence of pasteurized bone without remodeling. In the hopes of guaranteeing the most favorable results, the establishment of an animal model has been urgently awaited.

Bone clinically affected by a malignant bone tumor is usually resected accompanied by the surrounding muscle tissue, namely wide-resection. In the current study, as a basic priority, we established a model of pasteurized bone graft in rats, in which the graft was accompanied by resection of the surrounding muscle. Some surgeons utilize a method of covering the grafted bone with surrounding muscle in the expectation of a profitable clinical result. The benefit of muscle coverage seems to be supported by previous research showing the positive role of muscle stem cells in the bone repair process [11] and bone revascularization in musculocutaneous flaps [12].

Fibroblast growth factor (FGF) is a family of growth factors that control the proliferation and differentiation of various types of cells. FGF-2, or basic FGF, is a potent mitogen for osteoprogenitor cells, and it plays an important role in bone metabolism and in the regulation of osteoblastic cell proliferation and differentiation [13-16]. Furthermore, FGF-2 also plays an important role in osteoclastogenesis and angiogenesis [17].

In the current study, during the course of the establishment of a pasteurized bone model in rats, a preliminary analysis of the effect of the presence of muscle-covering to the pasteurized bone graft or the application of FGF-2 to pasteurized bone was carried out in terms of bone incorporation and bone absorption.

## Materials and methods

### Animals

Nine-week-old male Wistar rats (Kyudo Co. Ltd., Saga, Japan), ranging in weight from 300 g to 350 g, were used. The rats were kept at 22°C with free access to standard rat chow and water on a twelve-hour light-and-dark cycle. The current research was approved by the **E**thical **A**nimal **C**ommittee within Kyushu University (18-001-0).

### Surgical technique

We used an intramedullary fixation method to stabilize the grafted bone [18]. The rats were anesthetized with an intraperitoneal injection of Nenbutal (50 mg/kg; pentobarbital sodium). The rear leg was shaved and disinfected with povidone-iodine. After anesthetization was confirmed, a median parapatellar skin incision extending to the medial thigh was made. The femur was reached through an incision into the knee joint capsule and through the vastus medialis muscle. The patella was retracted laterally with the proximal muscle over the femur, then the surface of the femur was revealed.

The distal cut-line of the intercalary metaphyseal bone of the femur was designed above the epicondylar line. The length of the graft was sized to between 8 mm and 10 mm using an electronic bone saw, while protecting the posterior vessels. The graft was pasteurized in a sterile test-tube at 60°C for 30 min [2] in a Heat Block. In the groups receiving FGF-2 application, the pasteurized bone was soaked with human recombinant FGF-2 solution (250 μg/2.5 ml; Kaken Pharmaceutical Co., Ltd., Tokyo, Japan) for 30 min prior to reimplantation. The grafts were divided into 3 groups. The retracted anterior thigh muscle was repaired and used to cover the pasteurized bone without the application of FGF-2 (muscle covered [MC] +; FGF2-; 12 grafts), or the retracted anterior thigh muscle was removed, and sutured with pylorine to the residual muscle so as not to cover the graft, and either FGF-2 was not applied to the graft (MC-; FGF2-; 12 grafts) or FGF-2 was applied to the graft (MC-; FGF2+; 16 grafts).

Kirschner wire of 1.1 mm in diameter was inserted from an intercondylar area of the knee joint into the medullary space with a hand-held drill [18]. The wire was inserted until the wire penetrated as far as the proximal end of the femur, and stability was gained without disturbing the hip movement. The distal end of the Kirschner wire was cut, so as not to interfere with knee movement. After being washed with saline, the skin was sutured with pylorine.

### Radiographical evaluation of the bone formation, bone incorporation and bone absorption

Rats of the 3 groups were sacrificed at 2, 4 or 12 weeks under the same procedure as for anesthetization, but with massive dosage. These time points were chosen according to previous studies dealing with pasteurized bone grafts [1,3]. Each group included 4 grafts, except for the (MC-; FGF2+) group, which included 8 grafts. The femur with the reimplanted pasteurized graft was sampled, together with the surrounding soft tissue. Bone formation, bone incorporation and bone absorption were analyzed on the anterior portion of the proximal interface between the host and graft bone of the harvested samples radiographically.

Bone formation on the host bone was assessed. When the new bone formation was larger than the nearby cortex, the bone formation was classified as positive. In accordance with a previous study [18], the size of the bone formation was also quantitatively measured in the lateral view using Alpha Ease FC software (Alpha Innotech, San Leandro, CA, USA). The area was calculated in relation with that in the (MC-; FGF2-) group at 2 weeks in ratio. Bone incorporation, continuity between the graft and host bone, was assessed on either plain radiographs or histologically.

Bone absorption and formation on the graft were assessed with plain radiographs. When the bone was absorbed within the cortex, the result was classified as mild absorption, but when the cortex disappeared because of the absorption, the result was classified as severe absorption. In accordance with a previous study, we also used a score system regarding the status of the grafted bone in a modified way [1]. The appearance of the graft was scaled as follows: severe bone absorption (-2), mild bone absorption (-1), no change (0), single nodules of bone formation (1) and bridging or lamellar bone formation (2). An assessment of these results was made and agreed upon by AS, TY and NT.

### Tartrate-resistant acid phosphatase (TRAP) staining

After radiographical examination, the femurs with the graft were decalcified with EDTA (ethylenediaminetetraacetic acid), and cut sagittally, then stained with hematoxylin and eosin and tartrate-resistant acid phosphatase (TRAP) staining in order to demonstrate the osteoclasts. Deparaffinized sections were incubated at 37°C in 0.1 M acetate buffer (pH 5) (Sigma, St Louis, MO, USA) containing 220 μM naphthol AS-MX phosphate/dimethyl formaldehyde solution (Sigma), 2 mM fast red violet LB salt (Sigma), 50 mM L-(+)-sodium tartrate (Sigma), and 1 M MgCl_2 _for 30 min. Sections were then counterstained with hematoxylin.

### Statistical analysis

The results were compared using the Chi-square test (Williams's correction) for qualitative data and the Mann-Whitney *U*-test for quantitative data. A p value of < 0.05 was considered to indicate statistical significance.

## Results

**Figure 1:**
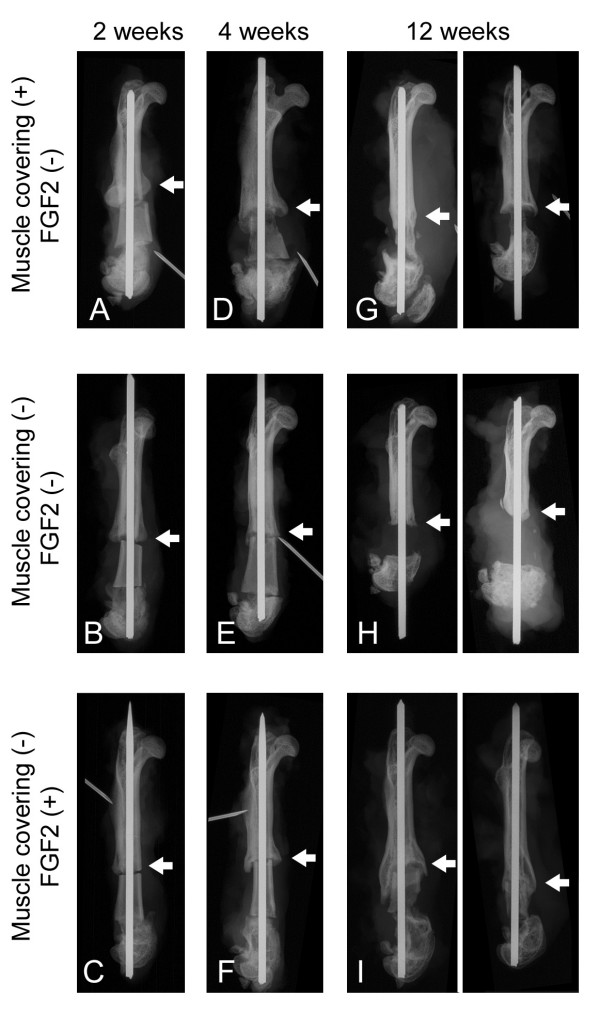
**Representative plain radiographs of pasteurized bone grafts at 2 weeks (A-C), 4 weeks (D-F) and 12 weeks (G-I) after grafting are shown**. Pasteurized bone with (MC+; FGF2-) (A, D, G), (MC-; FGF2-) (B, E, H) and (MC-; FGF2+) (C, F, I) is shown. Arrows show the anterior portion of the proximal interface between grafted bone and host bone for observation. Bone formations at the edge of the host bone can be seen in all 3 groups (A-C). Mild bone absorption can be observed on the (MC+; FGF2-) graft at 4 weeks (D). Massive bone absorption can be observed on the (MC+; FGF2-) graft (G, right) and on the (MC-; FGF2-) graft (H) at 12 weeks. Bone incorporation with a bridge of bone formation from the host bone can be seen on the (MC-; FGF2+) graft (I).

**Figure 2:**
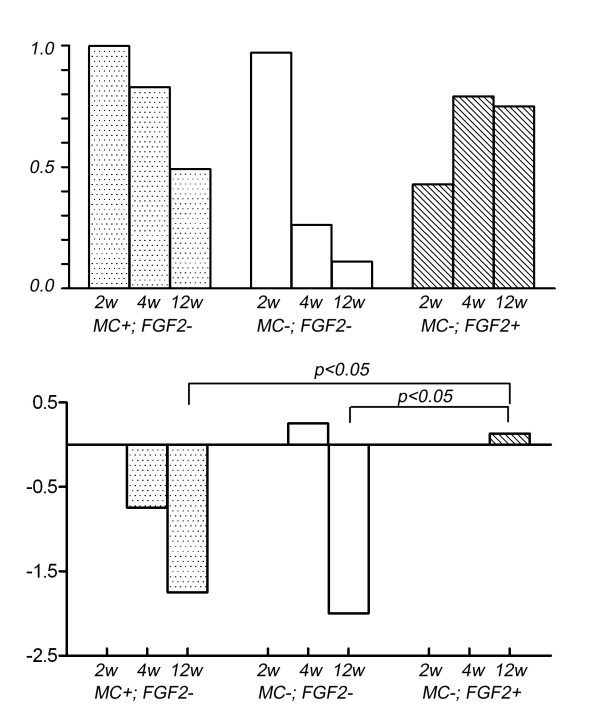
**The size of the bone formation of the host bone was also quantitatively measured in the lateral view**. The area was calculated in relation with that in the (MC-; FGF2-) group at 2 weeks in ratio (top). The status of the grafted bone is scaled and the average is given. The scale is as follows: severe bone absorption (-2), mild bone absorption (-1), no change (0), single nodules of bone formation (1) and bridging or lamellar bone formation (2) (bottom).

**Figure 3:**
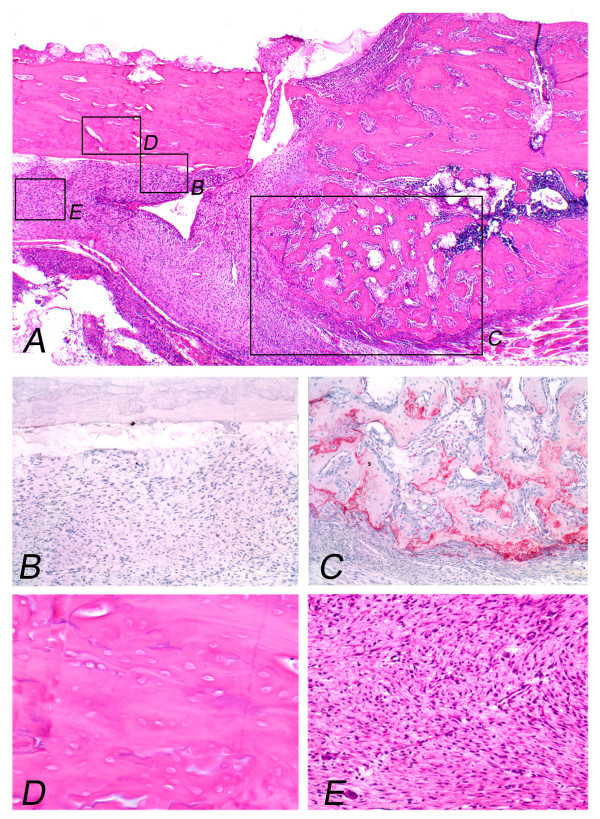
**Pasteurized bone with (MC-; FGF2-) at 2 weeks after grafting shows the grafted bone (left part) and the host bone (right part)**. Protuberant bone formation from the end surface of the host bone can be seen (A). Osteoclasts can not be observed on the surface of the grafted bone (B), whereas osteoclasts can be observed on the surface of the bone formation with numerous osteoclasts (C). Pasteurized bone shows empty lacunae without osteocytes (D). Pasteurized bone is surrounded by fibrous tissue (E). (Original magnification, H&E staining; A; ×70, D; E; ×250, TRAP staining; B; C; ×150).

**Figure 4:**
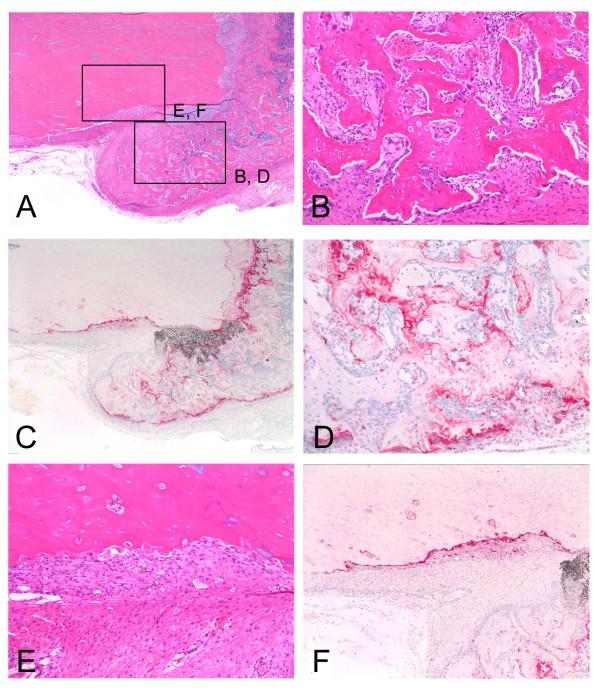
**Pasteurized bone with (MC+; FGF2-) at 4 weeks after grafting shows the grafted bone (left part) and the host bone (right part)**. Bone formation at the end of the host bone is rather mature (A, B) with osteoclasts on the surface of the bone trabeculae (C, D). Grafted bone characterized by empty lacunae is absorbed and replaced by fibrous tissue (E) associated with osteoclasts on the surface of the pasteurized bone (F). (Original magnification, H&E staining; A; ×70, B; E; ×150, TRAP staining; C; ×70, D; F; ×150).

**Figure 5:**
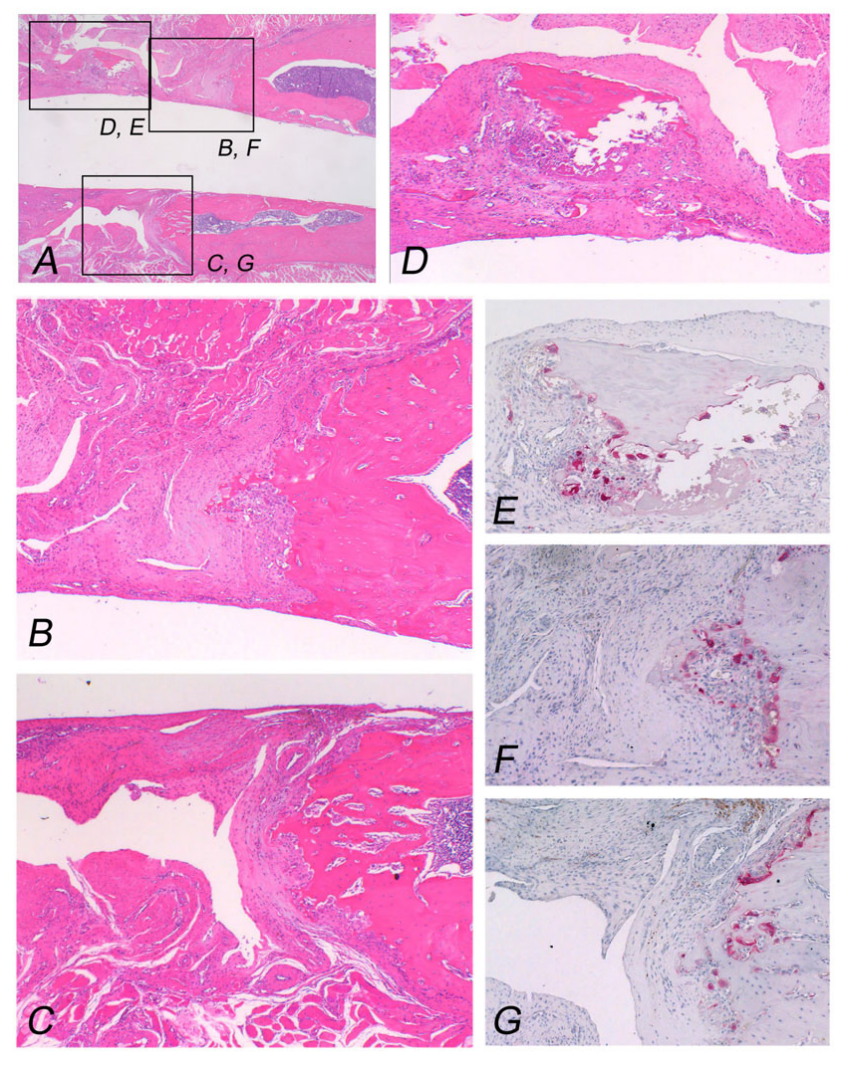
**Pasteurized bone with (MC+; FGF2-) at 12 weeks after grafting shows the grafted bone (left part) and the host bone (right part)**. Completely absorbed pasteurized bone has been replaced by fibrous tissue (A, B, C). The residual pasteurized bone with empty lacunae is embedded in the fibrous tissue (D). Osteoclasts can be seen on the residual bone (E) and the surface of the host bone (F, G). (Original magnification, H&E staining; A; ×70, B; C; D; ×100, TRAP staining; E; F; G; ×150).

**Figure 6:**
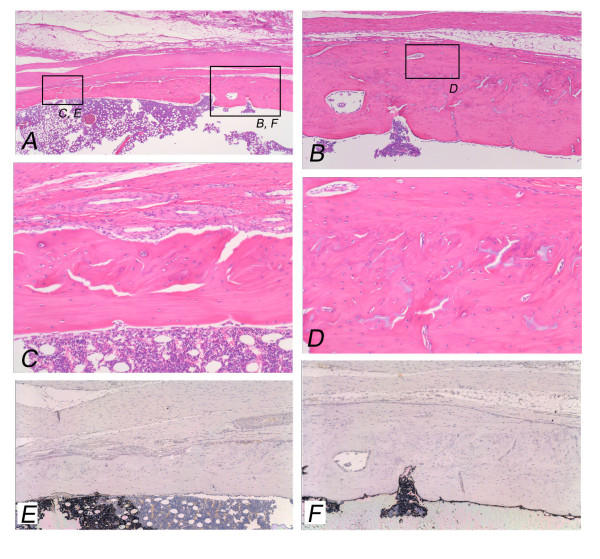
**Pasteurized bone with (MC-; FGF2+) at 12 weeks after grafting shows the grafted bone (left part) and the host bone (right part)**. Completely incorporated pasteurized bone to the host bone can be seen (A). Pasteurized bone has been remodeled with irregular bone matrix and osteocytes (B-D). Bone marrow formation can be seen (A, C). Osteoclasts can not be observed on the surface of the pasteurized bone (E) or the host bone (F). (Original magnification, H&E staining; A; ×70, B; ×150, C; D; ×190, TRAP staining; E; F; ×150).

Representative radiographs (Fig. 1) are shown. The summary results of bone formation, incorporation and absorption are shown in Tables 1 and 2, and in the graph of Figure 2. Representative histological appearance (Figs. 3, 4, 5 and 6) is also shown.

**Table 1:** Summary of bone formation, incorporation and absorption

	2 weeks	4 weeks	12 weeks
Bone formation on the host bone			
Muscle cover (+); FGF2 (-)	4/4 (100%)	4/4 (100%)	2/4 (50%)
Muscle cover (-); FGF2 (-)	3/4 (75%)	2/4 (50%)	0/4 (0%)^a^
Muscle cover (-); FGF2 (+)	3/4 (75%)	4/4 (100%)	5/8 (63%)^a^
			
Bone incorporation			
Muscle cover (+); FGF2 (-)	0/4 (0%)	2/4 (50%)	3/4 (75%)^b^
Muscle cover (-); FGF2 (-)	0/4 (0%)	0/4 (0%)	0/4 (0%)^b^
Muscle cover (-); FGF2 (+)	0/4 (0%)	2/4 (50%)	3/8 (38%)
			
Bone absorption on grafted bone			
Muscle cover (+); FGF2 (-)	0/4 (0%)	3/4 (75%)*	4/4 (100%)
		Mild 3	Mild 1
		Severe 0	Severe 3
			
Muscle cover (-); FGF2 (-)	0/4 (0%)	0/4 (0%)	4/4 (100%)
			Mild 0
			Severe 4
			
Muscle cover (-); FGF2 (+)	0/4 (0%)	0/4 (0%)	2/8 (25%)**
			Mild 1
			Severe 1

FGF; fibroblast growth factor.

^a^; p < 0.05, ^b^; p < 0.05

*; p < 0.05, **; p < 0.01 (compared to the other groups).

**Table 2:** Size and scores of bone formation and absorption

	2 weeks	4 weeks	12 weeks
^a^Relative size of bone formation on the host bone			
Muscle cover (+); FGF2 (-)	1.03	0.85	0.51
Muscle cover (-); FGF2 (-)	1	0.27	0.11
Muscle cover (-); FGF2 (+)	0.44	0.82	0.77
			
Status of grafted bone based on average score			
Muscle cover (+); FGF2 (-)	0.0	-0.75	-1.75

Bridging or lamellar bone (2)	0	0	0
Single nodules of bone (1)	0	0	0
No change (0)	4	1	0
Mild bone absorption (-1)	0	3	1
Severe bone absorption (-2)	0	0	3
			
Muscle cover (-); FGF2 (-)	0.0	0.25	-2.0

Bridging or lamellar bone (2)	0	0	0
Single nodules of bone (1)	0	1	0
No change (0)	4	3	0
Mild bone absorption (-1)	0	0	0
Severe bone absorption (-2)	0	0	4
			
Muscle cover (-); FGF2 (+)	0.0	0.0	0.13*

Bridging or lamellar bone (2)	0	0	1
Single nodules of bone (1)	0	0	2
No change (0)	4	4	3
Mild bone absorption (-1)	0	0	1
Severe bone absorption (-2)	0	0	1

FGF; fibroblast growth factor. The scores are shown in parentheses.

^a^; Relative size of bone formation to [muscle cover (-); FGF2 (-)] at 2 weeks on the host bone in ratio.

*; p < 0.05 (compared to the other groups).

### Two weeks after bone grafting

Plain radiographs at 2 weeks after grafting showed no prominent bone formation or bone absorption on the pasteurized bone, although prominent bone formation was observed at the host-bone edge (Figs. 1A, B, C). Prominent bone formation was seen in all 4 cases of the (MC+; FGF2-) group (4/4; 100%), in 3 out of 4 cases of the (MC-; FGF2-) group (3/4; 75%) and in 3 out of 4 cases of the (MC-; FGF2+) group (3/4; 75%) (Table 1). The average area of bone formation was 1.03, 1.0 and 0.44 in the (MC+; FGF2-), (MC-; FGF2-) and (MC-; FGF2+) groups, respectively (Table 2) (Fig. 2, top). On plain radiographs, neither bone incorporation nor bone absorption was observed in the series of 3 groups [(MC+; FGF2-) (0/4; 0%), (MC-; FGF2-) (0/4; 0%) and (MC-; FGF2+) (0/4; 0%)] (Table 1). No bone formation or absorption was seen on the grafted bone in any of the three groups. The average score of bone formation and bone absorption on the grafted bone was 0.0 (no change, 4 cases), 0.0 (no change, 4 cases) and 0.0 (no change, 4 cases) in the (MC+; FGF2-), (MC-; FGF2-) and (MC-; FGF2+) groups, respectively (Table 2) (Fig. 2, bottom). Histologically, protuberant bone formation with irregular bone trabeculae was seen at the edge of the host bone (Fig. 3A). Osteoclasts were not observed on the surface of the grafted bone (Fig. 3B), whereas osteoclasts were observed on the surface of the bone formation (Fig. 3C). The pasteurized grafts had empty lacunae lacking osteocytes throughout the entire area, suggesting a successful model of pasteurized bone graft (Fig. 3D). Pasteurized bone was surrounded by fibrous tissue (Fig. 3E). These findings were the same among the 3 groups, regardless of whether there had been muscle-covering or the application of FGF-2.

### Four weeks after bone grafting

On plain radiographs at 4 weeks after grafting, bone formation at the edge of the host bone was still frequently seen in all 3 groups [(MC+; FGF2-) (4/4; 100%), (MC-; FGF2-) (2/4; 50%), and (MC-; FGF2+) (4/4; 100%)] (Table 1) (Figs. 1D, E, F). The average area of bone formation was 0.85, 0.27 and 0.82 in the (MC+; FGF2-), (MC-; FGF2-) and (MC-; FGF2+) groups, respectively. The size of the bone formation was decreased in the (MC+; FGF2-) and (MC-; FGF2-) groups at 4 weeks compared with that at 2 weeks, with a prominent decrease in the (MC-; FGF2-) group (Table 2) (Fig. 2, top). Histologically, the bone formation was composed of rather regular bone trabeculae (Figs. 4A, B). Osteoclasts were also placed on the surfaces of the bone trabeculae (Figs. 4C, D). These histological features were consistent in all 3 groups. Bone incorporation was observed on either plain radiographs or histological specimens in half the cases in the (MC+; FGF2-) group (2/4; 50%) and in half the cases in the (MC-; FGF2+) group (2/4; 50%). Bone incorporation was not observed in any of the (MC-; FGF2-) cases (0/4; 0%). Bone absorption was seen in 3 out of 4 of the (MC+; FGF2-) cases (3/4; 75%). On the other hand, bone absorption was not observed in any of the (MC-; FGF2-) cases (0/4; 0%) or the (MC-; FGF2+) cases (0/4; 0%) (Table 1) (P < 0.05). These degrees of absorption on the (MC+; FGF2-) cases were within the cortex and were classified as mild (Table 1). Histologically, the absorbed pasteurized bone was replaced by fibrous or granulation tissue (Fig. 4E) associated with an accumulation of osteoclasts (Fig. 4F). The average score of bone formation and bone absorption on the grafted bone was -0.75 (mild bone absorption, 3 cases; no change, 1 case), 0.25 (no change, 3 cases; single nodules of bone formation, 1 case) and 0.0 (no change, 4 cases) in the (MC+; FGF2-), (MC-; FGF2-) and (MC-; FGF2+) groups, respectively (Table 2) (Fig. 2, bottom).

### Twelve weeks after bone grafting

On plain radiographs at 12 weeks after grafting, the number of cases with bone formation at the host bone became small in comparison to that at 2 or 4 weeks after grafting (MC+; FGF2-) (2/4; 50%), (MC-; FGF2-) (0/4, 0%), and (MC-; FGF2+) (5/8; 63%)] (Table 1) (Figs. 1G, H, I). Bone incorporation of the pasteurized bone to the host bone was seen in 3 out of 4 cases in the (MC+; FGF2-) group (3/4; 75%), but in only 3 out of 8 cases in the (MC-; FGF2+) group (3/8; 38%). On the other hand, bone incorporation was not observed in any of the (MC-; FGF2-) cases (0/4; 0%) with a significant difference to the (MC+; FGF2-) group (3/4; 75%) (Table 1) (P < 0.05). The average area of bone formation was 0.51, 0.11 and 0.77 in the (MC+; FGF2-), (MC-; FGF2-) and (MC-; FGF2+) groups, respectively (Table 2) (Fig. 2, top). The bone formation was particularly decreased in the (MC-; FGF2-) group at 12 weeks compared with the same group at 2 weeks. However, in the (MC+; FGF2-) cases, bone absorption was prominent (4/4; 100%), with the degree of absorption being classified as severe in 3 cases and mild in 1 case, whereas in the (MC-; FGF2+) cases, bone absorption was less prominent, in 2 out of the 8 cases (2/8; 25%) (P < 0.01), with the degree of absorption being classified as severe in 1 case and as mild in 1 case (Table 1). In the (MC-; FGF2-) cases, most of the pasteurized bone was almost completely absorbed (4/4; 100%) (Table 1) (Figs. 1G, H, I). Histologically, completely absorbed pasteurized bone was replaced by fibrous or granulation tissue (Figs. 5A, B, C). Osteoclasts were seen on the residual pasteurized bone which had empty lacunae without osteocytes (Figs. 5D, E) and on the surface of the host bone (Figs. 5F, G). On the other hand, pasteurized bone which had been completely incorporated to the host bone in one of the (MC-; FGF2+) cases showed an unclear interface between the pasteurized bone and the host bone (Fig. 6A). Bone matrix had been remodeled in an irregular fashion (Figs. 6B–D), and osteocytes could be observed on pasteurized bone (Fig. 6C) and on the host bone (Fig. 6D). Bone marrow formation was also observed (Figs. 6A, C). Osteoclasts were not observed on the surface of the pasteurized bone (Fig. 6E) or on the host bone (Fig. 6F). The average score of bone formation and bone absorption on the grafted bone was -1.75 (severe bone absorption, 3 cases; mild bone absorption, 1 case), -2.0 (severe bone absorption, 4 cases) and 0.13 (severe bone absorption, 1 case; mild bone absorption, 1 case; no change, 3 cases; single nodules of bone formation, 2 cases; bridging or lamellar bone formation, 1 case) in the (MC+; FGF2-), (MC-; FGF2-) and (MC-; FGF2+) groups, respectively. There was a significant difference between the (MC-; FGF2+) group and the other (MC+; FGF2-) and (MC-; FGF2-) groups (P < 0.05) (Table 2) (Fig. 2, bottom).

## Discussion

Heating of a resected bone segment at a low temperature, such as at 60°C for 30 min has been used as a method of pasteurization [3,4,19]. In the current study, pasteurized bone had empty lacunae at 2 weeks after grafting. For this reason, the pasteurized bone model seemed to be successful in terms of eliminating living cells, including osteocytes. Bone incorporation was seen in about half the cases of muscle-covering without FGF-2 at 4 weeks after the procedure. This result suggests that pasteurized bone after treatment at 60°C for 30 min helps to maintain bone induction ability.

Pasteurized bone without muscle-covering was examined as a model for wide resection of bone tumors. In a comparison between muscle-covering without FGF-2 and no muscle-covering without FGF-2, plain radiographs showed that after 2 weeks, bone was well formed at the edge of the hosted bone, and after 4 weeks, the size was decreased, especially when there was no muscle covering without FGF-2. Bone incorporation was seen in about half the (MC+; FGF2-) cases at 4 weeks after the procedure, whereas bone incorporation was seen in none of the 4 (MC-; FGF2-) cases. Therefore, muscle-covering of the pasteurized bone seemed to provide a positive effect on bone incorporation. Some surgeons utilize a method of covering a pasteurized bone graft using nearby muscle after resection of the affected bone together with the surrounding muscle. The current results showing an increased ability of bone incorporation with muscle-covering on the pasteurized bone seem to support the effectiveness of such clinical experience. The benefit of muscle coverage seems to be supported by previous research showing the positive role of muscle stem cells in the bone repair process [11]. Furthermore, it has been reported that the first step in bone formation in pasteurized bone might be the migration of mesenchymal stem cells from the contiguous normal medullary cavity [1]. The current study suggests that the circumstances outside the medullary cavity are also important for bone induction.

At 4 weeks after grafting, bone absorption of the pasteurized bone was only seen in the muscle-covering cases, and was not seen in cases without muscle-covering, with/without FGF-2. Bone absorption was replaced by granulation or fibrous tissue and was associated with osteoclast accumulation. At 12 weeks after grafting, in the series of (MC+; FGF2-) cases, even after bone incorporation in part, bone absorption of the pasteurized bone continued. Therefore, muscle-covering to pasteurized bone not only has a positive effect on bone incorporation to the host bone, but also on bone absorption associated with osteoclastic activity. In a previous model, a muscle flap was found to be superior to a cutaneous flap in revascularizing isolated bone segments, and furthermore, muscle flaps showed osteoblasts and osteoclasts, whereas neither were seen in the cutaneous flap [12]. In the current study, the increased positive effect on bone incorporation and bone absorption may be associated with the revascularizing that was associated with the surrounding muscle.

Mesenchymal stem cells are able to self-replicate and differentiate into a variety of cell types [20,21]. It has been suggested that FGF-2 increases the osteogenic and chondrogenic differentiation potentials of human mesenchymal stem cells [17]. Moreover, FGF-2 is a potent mitogen for osteoprogenitor cells, and it plays an important role in bone metabolism and in the regulation of osteoblastic cell proliferation and differentiation [13-16]. On the other hand, FGF-2 has been reported to stimulate bone resorption in bone organ cultures [22], as well as osteoclastogenesis in a mouse bone marrow culture [23]. FGF-2 plays a pivotal role in osteoclastogenesis through the up-regulation of RANKL (receptor activator of nuclear factor-kappa B ligand) [24]. In the case of FGF-2 application in the current study, achievement of bone incorporation was seen in 3 out of 8 (MC-; FGF2+) cases, while bone absorption was seen in only 2 out of these 8 cases. Considering that bone absorption was seen in all of the (MC-; FGF2-) cases, FGF-2 would seem to have a positive role to play in bone incorporation, and a negative role to play in bone absorption in the current model.

The lasting time of FGF-2 and its concentration from the grafted bone soaked in FGF-2 solution has been unknown. The possible releasing mechanism seemed to be a manner of diffusion. Since some research has reported that more than 80% of FGF-2 in solution form was cleared from the injected site of subcutaneous tissue of the mouse back within 1 day [25], it would seem that the effect of FGF-2 may be only short-term, even in the case of the current study. In a previous report on pasteurized bone, revascularization was thought to be important for bone remodeling [1]. FGF-2 also has angiogenic activity [26]. Therefore, it would seem that not only the initial induction of osteoblastic progenitor cells, but also the initial vascularization might play an important role in the process of bone incorporation of the pasteurized bone.

In this study, pasteurized bone grafts were soaked in FGF-2 solution and re-implanted. Results showing the potential usefulness of FGF-2 in the current study are encouraging with regard to pasteurized bone. A study including long-term use such as local delivery or controlled release of FGF-2 would be interesting, since the prolonged effect of FGF-2 may provide greater effectiveness in terms of increasing osteoblastic activity and decreasing bone absorption. In order to control the release of biologically-active growth factors, such as FGF-2, biodegradable hydrogels have been developed [25]. The effectiveness of the controled release of growth factors has been confirmed for the induction of angiogenesis in regenerated skin [26].

As for the limitations of this study, the current study did not include the group of muscle-covering pasteurized bone with the application of FGF-2 (MC+; FGF2+). During the establishment of a pasteurized bone model, we carried out a preliminary examination of the effect of the presence of muscle-covering or the application of FGF-2 to pasteurized bone as an independent concept. The synergetic effect of muscle-covering and FGF-2 administration is worth further examination. Due to the preliminary concept, the number of cases in each group was small and varied, yet the results seemed to be consistent in the current study. In any future project, a large number of cases with independent assessors would be preferable.

In the current study, we have assessed bone formation and bone absorption with plain radiographs. Histomorphometry analysis of the pasteurized bone grafts and the host bone to quantify the numbers of osteocytes, osteoclasts, osteoblasts and newly formed osteoid would be necessary to analyze bone remodeling. Moreover, proper markers would be helpful for visualizing blood vessel invasion or inflammatory cells within the granulation tissue surrounding the pasteurized bone, in order to analyze angiogenesis.

## Conclusion

In conclusion, we have established an animal pasteurized bone graft model in rats. Despite the small number of cases in each group, the results of each group suggest that muscle-covering without FGF-2 has an effect on bone incorporation, but is not able to prevent bone absorption to pasteurized bone. FGF-2 application seems to be useful in bone, in that it increases bone incorporation and prevents muscle absorption.

## Abbreviations

FGF: fibroblast growth factor; MC: muscle covered; TRAP: tartrate-resistant acid phosphatase

## Competing interests

The authors declare that they have no competing interests.

## Authors' contributions

AS drafted the manuscript. TY, AS, NT and KN performed the experiment. TY and AS participated in the design of the study. YI conceived of the study, and participated in its design and coordination and helped to draft the manuscript. All authors read and approved the final manuscript.
